# A Mobile Health Platform for Clinical Monitoring in Early Psychosis: Implementation in Community-Based Outpatient Early Psychosis Care

**DOI:** 10.2196/mental.8551

**Published:** 2018-02-27

**Authors:** Divya Kumar, Laura M Tully, Ana-Maria Iosif, Lauren N Zakskorn, Kathleen E Nye, Aqsa Zia, Tara Ann Niendam

**Affiliations:** ^1^ Department of Psychiatry and Behavioral Sciences University of California, Davis Sacramento, CA United States; ^2^ Division of Biostatistics Department of Public Health Sciences University of California, Davis Sacramento, CA United States

**Keywords:** mHealth, schizophrenia, smartphone, ecological momentary assessment, experience sampling

## Abstract

**Background:**

A growing body of literature indicates that smartphone technology is a feasible add-on tool in the treatment of individuals with early psychosis (EP) *.* However, most studies to date have been conducted independent of outpatient care or in a research clinic setting, often with financial incentives to maintain user adherence to the technology. Feasibility of dissemination and implementation of smartphone technology into community mental health centers (CMHCs) has yet to be tested, and whether young adults with EP will use this technology for long periods of time without incentive is unknown. Furthermore, although EP individuals willingly adopt smartphone technology as part of their treatment, it remains unclear whether providers are amenable to integrating smartphone technology into treatment protocols.

**Objective:**

This study aimed to establish the feasibility of implementing a smartphone app and affiliated Web-based dashboard in 4 community outpatient EP clinics in Northern California.

**Methods:**

EP individuals in 4 clinics downloaded an app on their smartphone and responded to daily surveys regarding mood and symptoms for up to 5 months. Treatment providers at the affiliated clinics viewed survey responses on a secure Web-based dashboard in sessions with their clients and between appointments. EP clients and treatment providers filled out satisfaction surveys at study end regarding usability of the app.

**Results:**

Sixty-one EP clients and 20 treatment providers enrolled in the study for up to 5 months. Forty-one EP clients completed the study, and all treatment providers remained in the study for their duration in the clinic. Survey completion for all 61 EP clients was moderate: 40% and 39% for daily and weekly surveys, respectively. Completion rates were slightly higher in the participants who completed the study: 44% and 41% for daily and weekly surveys, respectively. Twenty-seven of 41 (66%) EP clients who completed the study and 11 of 13 (85%) treatment providers who responded to satisfaction surveys reported they would continue to use the app as part of treatment services. Six (15%; 6/41) clients and 3 providers (23%; 3/13) stated that technological glitches impeded their engagement with the platform.

**Conclusions:**

EP clients and treatment providers in community-based outpatient clinics are responsive to integrating smartphone technology into treatment services. There were logistical and technical challenges associated with enrolling individuals in CMHCs. To be most effective, implementing smartphone technology in CMHC EP care necessitates adequate technical staff and support for utilization of the platform.

## Introduction

Utilizing smartphone technology to record real-world experiences as a supplement to mental health treatment can aid with insight and symptom management [1-3]. Treatment providers’ engagement with client-level information collected via smartphone and mobile health (mHealth) technology has the potential to provide useful insights regarding real-time symptoms that may differ from what clients express in session [4]. This integration of smartphone data with treatment services is particularly relevant in the context of psychotic illness, in which knowledge of current symptom severity is critical for rapid symptom and relapse management support [4,5].

Previous studies demonstrate that individuals with psychosis are amenable to incorporating smartphone technology into treatment and demonstrate high compliance [6-12]. Preliminary research also suggests that treatment providers will integrate information generated from smartphone technology into treatment plans [13]. Given the importance of early intervention in the treatment of psychotic disorders [14,15], focusing on integrating novel technological interventions with younger individuals experiencing psychosis is warranted. Our previous work indicates early psychosis (EP) individuals (ie, individuals within the first 3 years of psychosis onset or individuals at high risk for developing psychosis) are amenable to responding to daily surveys via smartphone app for up to 14 months, have high compliance rates, and provide self-report symptom data that are consistent with gold-standard clinician assessments [10].

Despite this growing body of research supporting the use of smartphone technology in the treatment of psychosis, 3 key questions remain. First, it is unclear whether consistent use of smartphone technology would be viable in outpatient clinics that are unaffiliated with a research center. These include community mental health centers (CHMCs) that are predominantly supported by federal and state funds (eg, Medicaid), and private-pay or insurance-based outpatient clinics. These types of clinics provide care to the majority of individuals with psychosis, not research-based programs. Implementation of smartphone technology in community-based care in the absence of an established research infrastructure is the next step in the dissemination of smartphone technology. Second, it is uncertain whether providers will be open to implementing this new technology into current treatment approaches. Provider integration is crucial to the successful implementation of mHealth technology into the broader scope of behavioral health care; without provider buy-in, even the best smartphone and mHealth platforms will flounder. Third, it is important to determine whether participants will continue to engage with this technology for long periods without being reimbursed for survey responses.

This study addresses these issues by testing the feasibility of implementing an mHealth platform as an add-on treatment tool in 4 community-based outpatient EP programs in Northern California that range from private-pay/insurance-based clinics to CMHCs. Individuals enrolled in treatment completed daily and weekly surveys assessing symptoms via a smartphone app for up to 5 months. Treatment providers then viewed client survey responses on a secure Web-based dashboard between appointments and in treatment sessions. Providers also completed surveys regarding feasibility and acceptability. We hypothesized the following: (1) EP clients would show high enrollment and low dropout, as well as high satisfaction and endorsement of continued use of the app as part of their treatment; (2) EP clients would show high survey completion in the absence of monetary incentives; and (3) treatment providers would endorse high utilization and acceptability of incorporating the technology into treatment. This paper establishes a protocol for implementing a smartphone app and affiliated Web-based dashboard as a supplement to treatment in EP care and details difficulties in scheduling, implementation, and technological challenges.

## Methods

### Setting

Treatment providers and EP clients were recruited from 4 EP clinics in Northern California: the Aldea Solano Supportive Outreach and Access to Resources (SOAR) program—a county-contracted CMHC supported by state and federal funds to provide services to residents of Solano County aged 12-25 years, regardless of insurance status; the Aldea Napa SOAR program—a CMHC funded by private donor money, state and federal funds, and private insurance to provide services to residents of Napa County aged 8-25 years; and the University of California (UC) Davis Early Psychosis Program, comprising 2 clinics embedded in the university setting: the Early Detection and Preventative Treatment (EDAPT) clinic, a self-pay or insurance-based clinic for individuals aged 12-40 years, regardless of county of residence, and the SacEDAPT clinic, a county-contracted clinic supported by federal and state funds, which provides care to Sacramento County residents aged 12-30 years, regardless of insurance status or ability to pay. All clinics provide coordinated specialty care [16] services to adolescents and young adults with EP, including individuals at clinical high risk for psychosis (ie, individuals displaying clinically significant but attenuated psychotic symptoms) and individuals within 2 years of their first psychotic episode, either in the context of a primary mood disorder (eg, bipolar disorder with psychotic features, major depressive disorder with psychotic features) or primary psychotic disorder (eg, schizophrenia, schizoaffective disorder). The coordinated specialty care model emphasizes early intervention for individuals experiencing psychosis via comprehensive support from a variety of mental health providers, including psychiatrists, therapists, supported education and employment specialists, case managers, and peer and family advocates.

### Participants

Two groups of participants were enrolled: clients (EP individuals enrolled in treatment) and treatment providers (clinical and support staff providing direct treatment services to EP clients).

EP clients consisted of individuals receiving care at any of the 4 EP clinics. Eligibility criteria for EP clients mirrored those required for enrollment in the clinics, that is, all clients enrolled in the clinics were eligible for participation. Eligibility criteria were as follows: English fluency, aged between 12 and 30 years at enrollment, WASI (Wechsler Abbreviated Scale of Intelligence) IQ score greater than 70 [17], no current substance abuse or dependence, and engagement with the clinic (attending at least one appointment a month). Owning a smartphone was not a requirement for participation in the study. Those who did not have a smartphone were provided an Android phone by the study for the duration of their participation in the study (see later sections for details). Spanish language consent processes were implemented to enable Spanish-speaking guardians of minor participants to enroll their child in the study.

Treatment providers included therapists (both licensed practitioners and unlicensed trainees), case managers, psychiatrists, supported education and employment specialists, and family advocates. Therapists included social workers, marriage and family therapists, and clinical psychologists (with both PsyD and PhD terminal degrees).

### Recruitment

Flyers detailing the study were placed around the clinics and in welcome packets for new clients. Treatment providers were approached about participation first; EP clients whose treatment providers agreed to participate were then approached. Treatment providers also informed their clients about the research opportunity and provided information to those who were interested. Enrollment for the study was ongoing for 8 months in the UC Davis Early Psychosis Program clinics (December 2015-June 2016 and December 2016), 8 months in the Aldea Solano SOAR clinic (May 2016-December 2016), and 6 months in the Aldea Napa SOAR clinic (July 2016-December 2016).

### Smartphones

We provided Android smartphones with a T-Mobile cell plan including unlimited calls, text, and data, to participants who did not possess their own. Due to changes in the standard hardware costs and smartphone handsets available from T-Mobile, 4 different types of Android smartphones were used over the course of the study: the Kyocera Hydro Life, the Kyocera XTRM, the Samsung Core Prime, and the Samsung On5. All smartphones had 4G/LTE (fourth-generation long-term evolution) data capabilities.

### The LifeData System

EP clients and treatment providers used the LifeData system [18], a mobile technology suite comprising 2 parts: a secure Web-based provider dashboard and the smartphone app RealLife Exp. EP clients responded to individual survey sets, called “LifePaks,” via RealLife Exp and providers viewed these responses on the dashboard. LifePaks contained standard survey questions (see following sections). The RealLife Exp app was downloaded via the App Store (iPhone) or GooglePlay store (Android). EP clients chose a time to receive their survey and had up to 90 min to complete it. LifePaks could be sent to each EP client at a specified time that best suited the client’s daily schedule; in this study, clients chose a survey notification time between 5:00 PM and 10:30 PM so as to capture their survey responses at the end of their day. They were advised to align the time of their survey notification with the time of their nighttime medication (if applicable), to use it as a reminder.

**Figure 1:**
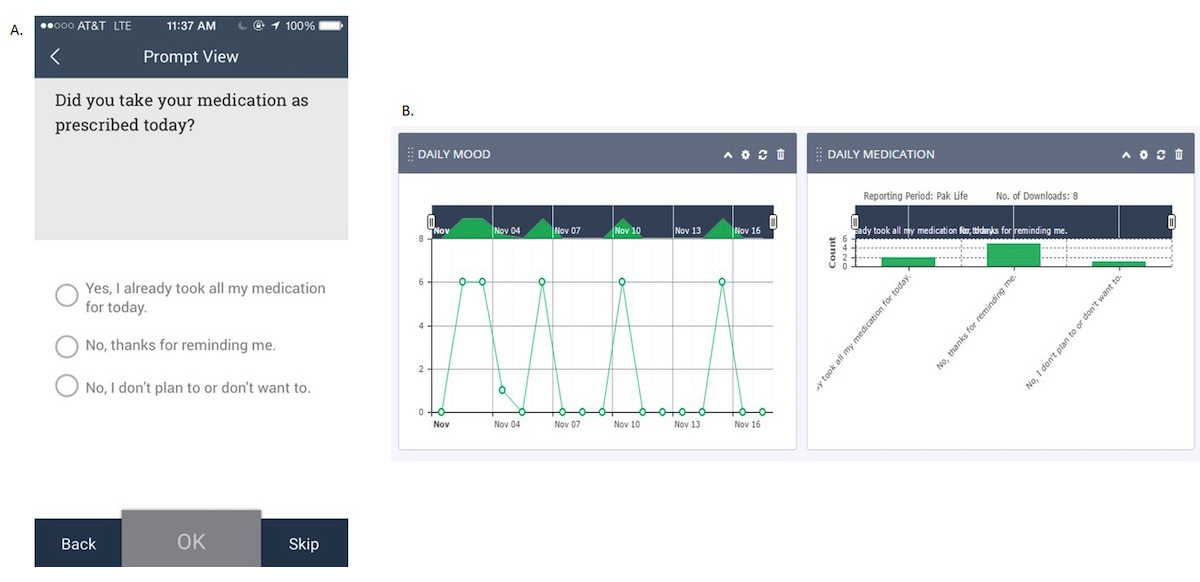
Screenshots of smartphone app and Web-based dashboard. (A) Example app view. EP clients responded to daily and weekly surveys in the app. Responses were summarized on the dashboard and discussed with treatment providers as part of regular clinic appointments. (B) Example dashboard view. Treatment providers could view plots of symptoms over time (daily mood and daily medication shown).

Responses to survey questions were displayed for treatment providers on the dashboard as a graph/chart, termed a “widget” in the LifeData System. Treatment providers could change the time frame of responses displayed to options ranging from the last 30 days, the last 7 days, the last 24 hours, or a custom period between 2 dates for specific questions of interest. Treatment providers could individualize the display of dashboards for each client. For example, if sleep was a symptom of interest for a particular client, a provider could move survey questions relating to amount of sleep to the top of the dashboard for quick viewing. EP clients were only able to review responses to survey questions on the dashboard during sessions with their treatment provider. See Figure 1 for illustrations of client app and treatment provider dashboard.

### Smartphone Surveys

**Figure 2:**
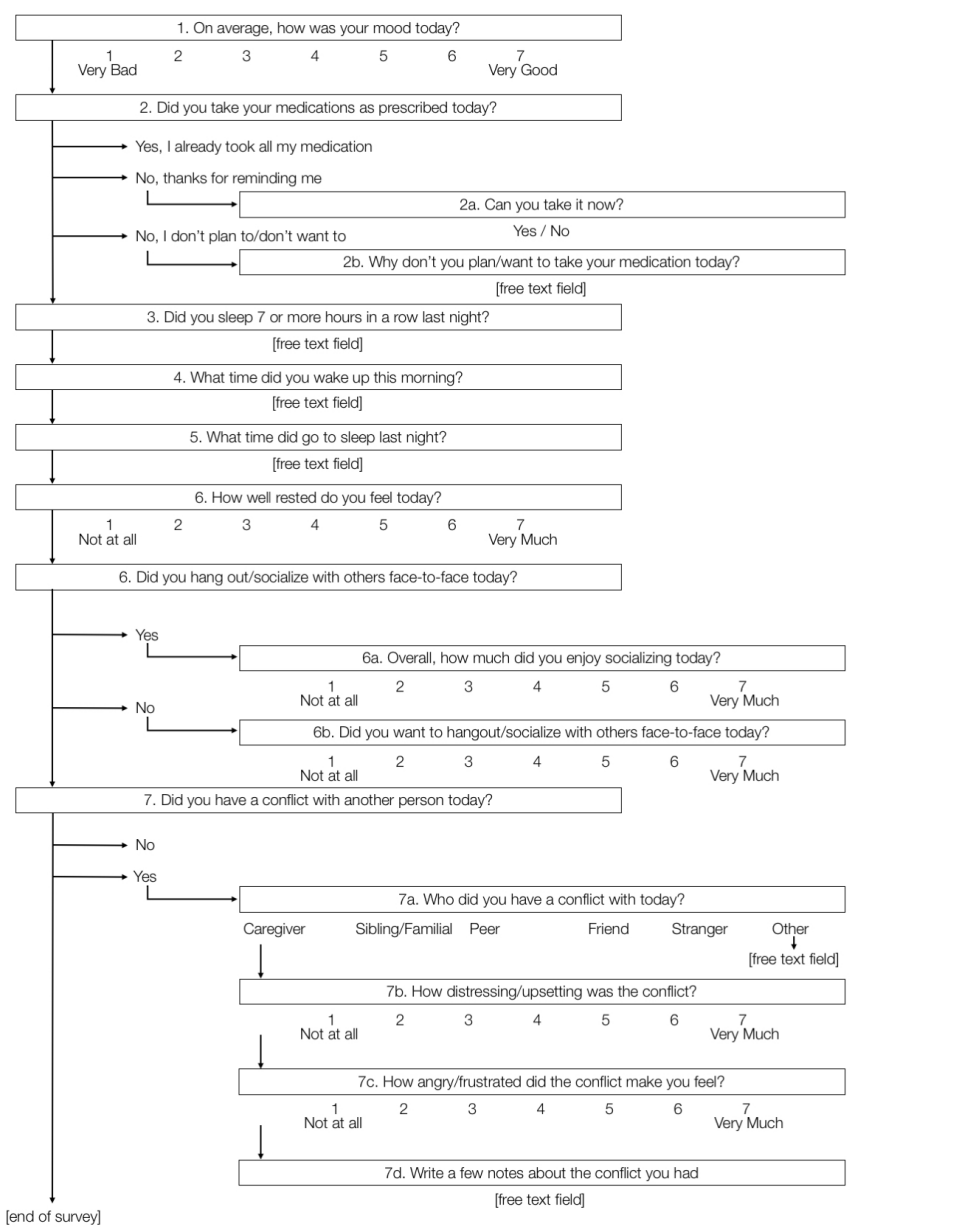
Daily survey questions. EP clients completed 7 to 14 questions daily between 5:00 PM and 10:30 PM. The daily survey took about 1 to 3 minutes to complete.

Daily and weekly surveys were sent via the app to EP clients at their chosen time between 5:00 PM and 10:30 PM. Survey questions were developed based on previous literature examining symptoms and functioning in individuals with schizophrenia-spectrum disorders [19-23] and have been implemented in our previous study testing the validity of gathering self-report symptom data via smartphone in EP populations [10]. The daily survey comprised questions pertaining to mood, medication use, socialization, and conflict; questions regarding medication use were removed for clients not taking medications. The weekly survey asked participants to rate on a 1-5 Likert scale (1=not at all, 3=half of the time, and 5=most of the time) how often in the past week they felt a range of symptoms. Surveys took approximately 1 to 3 minutes to complete. See Figure 2 for daily survey questions and Textbox 1 for weekly survey questions.

### Clinical Assessments and Self-Report Measures

Clients completed a series of self-report questionnaires and clinical assessments during the enrollment and study-end research appointments. Therapeutic alliance from the perspective of the EP client and treatment provider was obtained using the Scale to Assess Therapeutic Relationship [24]. Medication adherence was assessed via the Medication Adherence Rating Scale [25], and medication side effects were assessed using the Glasgow Antipsychotic Side-Effect Scale [26]. Client insight into clinical symptoms was measured via the Insight Scale [27]. Comfort with and utilization of smartphone/mobile technology in daily life was assessed using a Comfort with Technology questionnaire, modified from the Technology Readiness Index [28]. Drug and cannabis use were assessed using the Drug Use Screening Inventory [29] and the Cannabis Use Problems Identification Test [30], respectively.

Clinical symptoms were assessed by research staff at enrollment and study-end appointments using the Global Functioning Social and Role Scales [31,32], the Brief Psychiatric Rating Scale (BPRS) [33], and the Clinical Global Impression Scale [34]. BPRS item scores were summed to create composite symptoms scores for positive symptoms (7 items: grandiosity, unusual thought content, bizarre behavior, disorientation, hallucinations, suspiciousness, and conceptual disorganization), negative symptoms (3 items: blunted affect, motor retardation, and emotional withdrawal), depression/anxiety symptoms (4 items: depression, anxiety, suicidality, and guilt), and symptoms of agitation/mania (6 items: motor hyperactivity, excitement, distractibility, tension, uncooperativeness, and mannerisms and posturing) [35]. Good reliability for composite symptom scores has been demonstrated in prior publications [10]. BA-level research staff conducted assessments, supervised by licensed clinical psychologists (TAN and LMT). Staff were trained to good-to-excellent reliability (intra class correlations>.75) via independent ratings of 4 videotaped interviews.

Treatment providers (excluding psychiatrists) were asked to fill out weekly surveys regarding their utilization of the dashboard, either via a paper questionnaire or a Web-based survey using the Qualtrics platform (Qualtrics, Provo, UT).

Both EP clients and treatment providers filled out satisfaction surveys upon completion of the study, detailing their experiences using the app. Satisfaction survey questions asked about ease of use of the app, usefulness of responses, and any suggestions of potential changes for continued use.

### Procedures

EP clients enrolled in the study for up to 5 months and attended an assessment appointment at enrollment and study-end appointments. All study procedures were approved by the UC Davis Institutional Review Board.

At enrollment, EP clients completed all clinical assessments and questionnaires and downloaded the RealLife Exp app to their smartphone. They then created a RealLife Exp account, which was linked to a unique LifePak designed for them with agreed upon survey times. All clients completed a practice survey set on the phone with research staff at this appointment. EP clients who did not own their own phone set up a study-provided phone with the assistance of research staff.

Treatment providers were oriented to the visualization of responses on the LifeData dashboard during a 1-hour study enrollment session. Research staff demonstrated how to access the dashboard, what individual clients’ responses would look like, and how to interpret the different visualizations of survey responses. To ensure providers had an accurate understanding of the dashboard, they were asked to explain different responses to questions on the dashboard and to navigate through the process on their own at the end of the enrollment. Providers were also given a 1-page reference document that contained log-in information, how to customize dashboards, and solutions to common technical glitches. Upon completion of an EP client’s enrollment appointment, their treatment provider received access to the dashboard for that client. Treatment providers were instructed to review survey responses with clients during regular treatment sessions, as well as between appointments. Treatment providers were encouraged to use dashboard/survey data to prompt contact with clients between sessions per their clinical judgment or supervisor’s recommendations. Treatment providers were instructed to review survey data with participants solely in the context of treatment; treatment providers were not instructed to persuade people to do the study or complete surveys.

**Weekly survey questions. Participants completed 16 questions each Sunday via the app.**

This week I…

1. Felt sad or depressed
2. Felt anxious or worried
3. Felt cheerful or happy
4. Felt confused or distracted
5. Felt I had to be “on guard” with others, even my friends
6. Felt friendly or social
7. Felt lively or energetic
8. Felt like I did not care about anything
9. Felt unmotivated and could not get things done
10. Felt challenged and overwhelmed by my usual daily activities
11. Saw people, shadows, or things and then realized they were not really there
12. Felt hopeful about my future
13. Felt supported by my family and friends
14. Heard sounds, voices, or whispers but then realized there was nothing there
15. What percentage of your prescribed medication have you taken the past week? (0-100%)
16. Tell us about any other experiences you had this week (Response: free text entry)

EP clients were prompted once a day to answer a survey; on Sundays, they were prompted twice, once to answer the daily survey and once for the weekly survey. Research staff did not systematically engage with EP clients during their time in the study, with the exception of responding to technical errors with the app or the study-provided smartphone. These errors were commonly reported by the client or their treatment provider and were also occasionally noticed by a research staff member while exporting survey response data for analysis.

Consistent with Institutional Review Board guidelines, EP clients were compensated US $50 for completing the enrollment assessment and US $50 for completing the study-end assessment. EP clients were not reimbursed for completing surveys over the course of the study. Treatment providers were not compensated for taking part in the study.

Dashboards remained active until the clinical appointment following an EP client’s study-end appointment. The app was deleted from the client’s phone at study end. If the client borrowed a study phone, it was returned and reset to factory settings, removing all identifiable data. Study-owned smartphones were reused with new EP clients.

### Data Analysis

Descriptive statistics were used to summarize client and provider characteristics, study enrollment, daily and weekly survey completion, and length of time participants were enrolled in the app, as well as participant ratings on satisfaction and perceived impact on clinical care. Daily and weekly survey completion rates were calculated for each participant by summing the number of daily/weekly surveys completed and dividing this value by the total number of daily/weekly surveys they were sent during the entire period that participants were enrolled in app (ie, length of time in study). We examined differences in baseline symptom severity (baseline BPRS composite symptom scores) and survey completion between completers and noncompleters (ie, individuals who completed a study-end assessment appointment vs individuals who did not) using the Wilcoxon two-sample test. We used Spearman rank correlation coefficient (ρ) to examine relationships between baseline symptom severity, length of time in study, and survey completion rates, both in the overall sample and in the subset of participants who completed the study.

## Results

### Early Psychosis Client Enrollment

Of the 108 EP clients eligible across the 4 sites, 61 (56%) enrolled in the study. Specifically, 59% (16/27) of clients in the EDAPT clinic participated; 62% (28/45) of clients in the SacEDAPT clinic participated; 44% (11/25) of clients in the Aldea Solano SOAR clinic participated; and 55% (6/11) of clients in the Aldea Napa SOAR clinic participated. Lack of consent from individuals who did not enroll in the study precludes analyses of group differences between those who declined and those who enrolled. Demographic and clinical characteristics of EP clients are displayed in Table 1.

Forty-one EP clients (67%; 41/61) completed the study (“completers”): 33 completed all 5 months of data collection and 8 completed 3 to 4 months (including enrollment and study-end appointments) and were discontinued early due to the end of data collection in the grant-funded period. Twenty (33%; 20/61) EP clients did not finish the study (“noncompleters”): 18 (5 EDAPT, 9 SacEDAPT, 1 Napa, 3 Solano) were withdrawn from the study by research staff because they discontinued services with the clinic; 2 (2 EDAPT) withdrew from the study on their own due to lack of interest in continuing to participate. Average length of time in study for all 61 EP clients was 138 days (SD 49.2, range 25-232). Average length of time in study for the 41 completers was 156.2 days (SD 36.5, range 67-232) vs 100.5 days (SD 51.5, range 25-153) for the 20 noncompleters.

In the overall sample (n=61), length of time in study was not related to baseline positive (ρ=−0.03, *P*=.80), negative (ρ=0.02, *P*=.90), or depression/anxiety (ρ=−0.08, *P*=.55) symptoms, but was correlated with agitation/mania symptoms (ρ=0.25, *P*=.0495). A similar pattern was observed in the 41 completers, with no significant relationships between time in study and baseline positive (ρ=0.06, *P*=.69), negative (ρ=0.23, *P*=.15), or depression/anxiety (ρ=0.01, *P*=.97) symptoms, but a statistically significant correlation with agitation/mania symptoms (ρ=0.35, *P*=.03). There were no differences in positive (Wilcoxon two-sample test *P*=.58), negative (*P*=.24), or agitation/mania (*P*=.37) symptoms between study completers and noncompleters. Noncompleters showed more severe depression/anxiety symptoms at trend level (*P*=.08).

### Treatment Provider Enrollment

Demographics of treatment providers are displayed in Table 2. Of the 20 eligible treatment providers across all sites, 20 (100%) enrolled in the study. Twenty-five percent (5/20) of treatment providers provided care in both the EDAPT and SacEDAPT clinics, 5% (1/20) in the EDAPT clinic alone, 35% (7/20) in the SacEDAPT clinic alone, 5% (1/20) provided care in both the Aldea SOAR Solano and Napa programs, 20% (4/20) provided care in the Aldea SOAR Solano program alone, and 10% (2/20) provided care in the Napa SOAR Solano program alone.

**Table 1:** Demographic and clinical characteristics of enrolled early psychosis clients (N=61). Due to rounding, percentages might not sum to 100.

Characteristic		UC^a^ Davis EDAPT^b^ clinic (n=16)		UC Davis SacEDAPT clinic (n=28)		Napa Aldea SOAR^c^ clinic (n=6)		Solano Aldea SOAR clinic (n=11)	
Age (years), mean (SD)		18.6 (2.9)		17.5 (3.8)		17.5 (3.6)		15.1 (2.5)	
Education (years), mean (SD)		13.4 (2.0)		10.3 (2.8)		11.5 (4.9)		11.3 (1.2)	
Parental education (years), mean (SD)		13.8 (2.5)		12.7 (2.3)		12.5 (3.5)		14.0 (5.6)	
Male gender, n (%)		7 (44)		16 (57)		3 (50)		6 (55)	
**Race, n (%)**									
	African American		0 (0)		8 (29)		0 (0)		1 (9)
	Asian American		3 (19)		7 (25)		0 (0)		0 (0)
	White		9 (56)		8 (29)		2 (33)		3 (27)
	Native American		0 (0)		1 (4)		0 (0)		0 (0)
	Multiple/Other		3 (19)		4 (14)		4 (67)		7 (64)
Hispanic ethnicity, n (%)		5 (31)		3 (11)		4 (67)		7 (64)	
Loaned phone, n (%)		2 (13)		18 (64)		1 (17)		8 (73)	
**Type of phone, n (%)**									
	Android		11 (69)		25 (89)		3 (50)		8 (73)
	iPhone		5 (31)		3 (11)		3 (50)		3 (27)
**Diagnosis, n (%)**									
	Schizophrenia spectrum disorder		9 (56)		18 (64)		0 (0)		7 (64)
	Mood disorder with psychotic features		1 (6)		6 (21)		1 (17)		0 (0)
	Clinical high risk		6 (38)		4 (14)		5 (83)		4 (36)
**Baseline BPRS^d^ symptoms, mean (SD)**									
	Positive symptoms		13.4 (4.4)		13.5 (5.6)		14.2 (4.6)		13.1 (4.4)
	Negative symptoms		4.8 (1.7)		6.8 (2.9)		5.0 (3.1)		6.3 (2.6)
	Depression/anxiety symptoms		10.1 (4.3)		10.0 (5.0)		7.3 (3.8)		9.5 (4.8)
	Agitation/mania symptoms		8.3 (1.8)		10.3 (2.7)		8.5 (2.1)		8.1 (1.5)

^a^UC: University of California.

^b^EDAPT: Early Detection and Preventative Treatment.

^c^SOAR: Supportive Outreach and Access to Resources.

^e^BPRS: Brief Psychiatric Rating Scale.

**Table 2:** Demographic characteristics of enrolled early psychosis treatment providers (N=20). Due to rounding, percentages might not sum to 100.

Demographic characteristic		All treatment providers (n=20)	UC^a^ Davis EDAPT^b^ clinic (n=1)	UC Davis SacEDAPT clinic (n=7)	Napa Aldea SOAR^c^ clinic (n=2)	Solano Aldea SOAR clinic (n=4)	Multiple clinics (n=6)^d^
**Age group^e^, n (%)**							
	25-34	10 (56)	0 (0)	5 (83)	1 (50)	1 (25)	3 (60)
	35-44	4 (22)	0 (0)	0 (0)	1 50)	1 (25)	2 (40)
	45-54	2 (11)	0 (0)	1 (17)	0 (0)	1 (25)	0 (0)
	55-64	2 (11)	1 (100)	0 (0)	0 (0)	1 (25)	0 (0)
Male gender, n (%)		3 (15)	1 (100)	1 (14)	0 (0)	0 (0)	1 (17)
**Race, n (%)**							
	Asian American	4 (20)	0 (0)	1 (14)	0 (0)	1 (25)	2 (33)
	White	16 (80)	1 (100)	6 (67)	2 (100)	3 (75)	4 (67)
Hispanic ethnicity, n (%)		5 (25)	0 (0)	2 (29)	1 (50)	0 (0)	2 (33)
**Degree obtained, n (%)**							
	MFT^f^	6 (30)	0 (0)	1 (14)	2 (100)	1 (25)	2 (33)
	MSW^g^	3 (15)	0 (0)	0 (0)	0 (0)	2 (50)	1 (17)
	PsyD^h^	4 (20)	0 (0)	3 (43)	0 (0)	0 (0)	1 (17)
	PhD^i^	2 (10)	1 (100)	1 (14)	0 (0)	0 (0)	0 (0)
	MD^j^	4 (20)	0 (0)	2 (29)	0 (0)	0 (0)	2 (33)
	Other	1 (5)	0 (0)	0 (0)	0 (0)	1 (25)	0 (0)
Bilingual practitioner^k^, n (%)		9 (45)	0 (0)	4 (57)	1 (50)	1 (25)	3 (50)
Licensed practitioners, n (%)		11 (55)	1 (100)	2 (29)	1 (50)	1 (25)	6 (100)

^a^UC: University of California.

^b^EDAPT: Early Detection and Preventative Treatment.

^c^SOAR: Supportive Outreach and Access to Resources.

^d^Six treatment providers provided care in more than one clinic: 5 provided care in both the UC Davis SacEDAPT and EDAPT clinics; 1 provided care in both the Napa and Solano Aldea SOAR clinics.

^e^Frequency missing=2, 1 in the Multi clinics and 1 in SacEDAPT.

^f^MFT: Marriage and Family Therapist.

^g^MSW: Master’s in Social Work.

^h^PsyD: Doctor of Psychology.

^i^PhD: Doctor of Philosophy.

^j^MD: Medical Doctor.

^k^Bilingual practitioners were providers who spoke 1 or more languages fluently (in addition to English) and used them as part of the clinical practice with clients. Nine providers identified as bilingual practitioners, languages included were as follows: Mandarin (n=1), Punjabi (n=1), Spanish (n=5), Korean (n=1), and Turkish (n=1).

All treatment providers remained in the study from the point they were enrolled until either the end of data collection (n=10) or until they left their position as a provider at one of the affiliated clinics (n=10). Over the course of the study, 26% (16/61) of EP clients had a change in 1 or more of their treatment providers: 18% (11/61) had a change in their primary therapist only (8 in SacEDAPT, 1 in EDAPT, 2 in Aldea SOAR Solano), 3% (2/61) had a change in their psychiatrist only (2 in SacEDAPT), and 5% (3/ 61) had a change in both their primary therapist and their psychiatrist (3 in SacEDAPT). The average number of enrolled clients per treatment provider overall was 6.5 (SD 6.1, minimum=1, maximum=25). Therapists (n=16) had an average of 4.8 enrolled clients (SD 3.9, range 1-15); psychiatrists (n=4) had an average of 13.5 enrolled clients (SD 8.5, range 1-25).

### Survey Completion Rates

In the overall sample (n=61), average daily survey completion rate was 41% (SD 25%, median 41%, range 0-89%) and average weekly survey completion rate was 39% (SD 28%, median 40%, range 0-100%). Daily survey completion was not related to baseline positive (ρ=0.21, *P*=.11), negative (ρ=−0.09, *P*=.49), depression/anxiety (ρ=0.10, *P*=.43), or agitation/mania (ρ=−0.14, *P*=.27) symptoms. Similarly, weekly survey completion was not related to baseline positive (ρ=0.15, *P*=.24), negative (ρ=−0.15, *P*=.25), depression/anxiety (ρ=0.03, *P*=.83), or agitation/mania (ρ=−0.22, *P*=.09) symptoms. There were no differences in daily or weekly survey completion rates between EP clients who had their own smartphone (n=32) and EP clients who were given a study smartphone (n=29) (all *P* s>.3), indicating that the provision of a smartphone was not an incentive to complete surveys in and of itself.

Survey completion was higher in the 41 completers (mean daily 44% [SD 25%], median 44%, range 0-82%; mean weekly 42% [ SD 28%], median 46%, range 0-92%) compared with the 20 noncompleters (mean daily 35% [SD 25%], median 33%, range 0-89%; mean weekly 33% [SD 27%], median 31%, range 0-100%), but the difference did not reach statistical significance (*P*=.22 for daily and .15 for weekly). In the 41 completers, daily survey completion was not related to baseline symptoms (all *P* s>.1); however, weekly survey completion rates were related to baseline negative (ρ=−0.31, *P*=.047) and agitation/mania (ρ=−0.32, *P*=.04) symptoms, such that more severe symptoms were associated with lower weekly survey completion rates. No relationships between weekly survey completion and positive or depression/anxiety symptoms were observed in completers (all *P* s>.3).

### Treatment Provider Use of Dashboard

Treatment providers (n=16), excluding psychiatrists (because of time constraints), were asked to complete weekly surveys regarding their use of the dashboard in regular treatment sessions with enrolled clients. Seventy-five percent (12/16) of the treatment providers completed at least one survey over the course of the study; response rate was lower than expected due to the many competing demands placed on community-provider time, thus data should be viewed as preliminary and interpreted with caution. We summarized data after first averaging within provider. Treatment providers reported that they had treatment sessions with an average of 2 enrolled clients per week (2.0 [SD 2.1], range 1-8) and incorporated the dashboard into an average of 1 session per week (1.4 [SD 0.4], range 1.0-2.3). When they incorporated the dashboard as part of regular treatment sessions, providers reported discussing it for an average of 16% of session time (SD 9%, range 10-33%)—approximately 8-10 min of a standard 50-min session—and rated the dashboard data as moderately useful (on a 1-7 Likert scale) as a treatment enhancement tool (3.9 [SD 1.4], range 1.3-6.0).

### Satisfaction Surveys

All of the 41 EP clients that completed the study, as well as 13 of 16 treatment providers, completed surveys at the end of the study assessing satisfaction with the app and perceived effect of the app on treatment and behavior. Results of these surveys can be viewed in Tables 3-6. Of note, 66% (27/41) of EP clients stated they would continue to use the app as part of treatment services if it was made available and 61% (25/41) stated they would recommend the app to a friend. Thirty-seven percent (15/41) of EP clients found the app to be extremely helpful, 44% (18/41) found it to be a little helpful, and 20% (8/41) found it made no difference. Overall, 27% (11/41) of EP clients reported that the data provided by the app had at least some effect on their behavior (see Table 3). A small minority of clients (12%, 5/41) stated that they would not continue to use the app and 22% (9/41) reported that they might continue to use the app if it was better. Similarly, 15% (6/41) reported that they would not recommend the app to a friend and 24% (10/41) reported that they might recommend it if it was better. Satisfaction surveys from treatment providers included the following results: 85% (11/13) reported they would continue to use the app as part of treatment services, whereas 15% (2/13) reported that they might continue to use the app if it was better. Similarly, 85% (11/13) reported that they would recommend the app to a client, and 15% (2/13) reported they might recommend it if it was better. Fifty-four percent (7/13) stated the app was extremely helpful, 46% (6/13) stated it was a little helpful, and 0% (0/13) stated it made no difference. Finally, 15% (6/41) of EP clients and 23% (3/13) of treatment providers noted that technical glitches in the RealLife Exp platform caused frustration and limited engagement.

Fifty-one percent (21/41) of EP clients suggested improvements for the app (see Table 5): 15% (6/41) suggested improvements to the technical stability of the app (eg, reduce app crashes, improve reminder notification stability, facilitate easier sign-in after forced log-outs); 17% (7/41) suggested product enhancements to the app (eg, improved user interface, inclusion of data summaries and graphs in the app to track self-progress, online community engagement with peers); 32% (13/41) suggested improvements to the surveys (eg, increased number of questions regarding symptoms, more flexibility on when surveys must be completed during the day, wider range of response options to sliding scale questions); and 3% (1/41) suggested the treatment provider team should use the dashboard more in sessions to enhance care.

Thirty-eight percent (5/13) of treatment providers suggested improvements for the app and dashboard (see Table 6): 15% (2/13) suggested improvements to technical stability (eg, fix missing data on dashboard, fix glitches that prevented clients from completing surveys); 8% (1/13) suggested dashboard enhancements (more user-friendly graphs); 8% (1/13) suggested including rewards/badges for surveys to facilitate survey completion; and 8% (1/13) suggested making access to the dashboard easier to facilitate provider use in treatment sessions (eg, create a mobile app to access the dashboard for tablet/smartphone).

**Table 3:** Summary of early psychosis clients’ perceived effect of the use of surveys (N=41). Due to rounding, percentages might not sum to 100.

Survey questions	A lot, n (%)	A little, n (%)	Somewhat, n (%)	Not at all, n (%)
To what extent did RealLife Exp improve the quality of your treatment services?	10 (24)	12 (29)	13 (32)	6 (15)
Did RealLife Exp improve your relationship with your treatment team?	8 (20)	13 (32)	11 (27)	9 (22)
Did RealLife Exp help you understand your symptoms?	9 (22)	7 (17)	14 (34)	11 (27)
Did RealLife Exp help you and your treatment team improve your symptoms and overall well-being?	9 (22)	8 (20)	19 (46)	5 (12)
Did RealLife Exp help you remember to take your medication?	20 (49)	11 (27)	7 (17)	3 (7)
Did RealLife Exp help you manage your symptoms?	9 (22)	10 (24)	15 (37)	7 (17)
Did RealLife Exp help you feel more in control of your symptoms?	9 (22)	10 (24)	14 (34)	8 (20)
Are you more motivated to keep up with your symptom management and medication routine?	16 (39)	16 (39)	6 (15)	3 (7)

**Table 4:** Summary of early psychosis clients’ (N=41) and treatment providers’ (N=13) satisfaction surveys. Due to rounding, percentages may not sum to 100. Satisfaction data are missing from 7 treatment providers.

Survey questions		Early psychosis clients, n (%)	Treatment providers, n (%)
**How easy was it to use RealLife Exp?**			
	Extremely Easy	25 (61)	5 (38)
	Fairly Easy	15 (37)	8 (62)
	Somewhat Difficult	1 (2)	0 (0)
	Extremely Difficult	0 (0)	0 (0)
**How easy was it to complete the surveys on RealLife Exp?**			
	Extremely Easy	27 (66)	9 (69)
	Fairly Easy	13 (32)	4 (31)
	Somewhat Difficult	1 (2)	0 (0)
	Extremely Difficult	0 (0)	0 (0)

**Table 5:** Summary of the features that early psychosis clients desired in an app for early psychosis care (N=41). Due to rounding, percentages might not sum to 100.

Survey questions		n (%)
**Please circle ALL the features you would like from an application like RealLife Exp**		
	Connection to a community	14 (34)
	Connection to your care team	25 (61)
	Helpful information about symptoms	34 (83)
	Personal insights about your behavior	36 (88)
	Rewards and badges for survey completion	18 (44)
**Please circle the ONE feature you would like most from an application like RealLife Exp**		
	Connection to a community	3 (7)
	Connection to your care team	9 (22)
	Helpful information about symptoms	10 (24)
	Personal insights about your behavior	18 (44)
	Rewards and badges for survey completion	1 (2)

**Table 6:** Summary of the features that treatment providers desired in a mobile health platform for early psychosis care (N=41).

Survey questions		n (%)
**What feature(s) of LifeData did you find useful? Choose all that apply:**		
	Graphs of client daily symptoms	12 (92)
	Graphs of client weekly symptoms	10 (77)
	Information on medication habits of clients	12 (92)
	Information on sleeping habits of clients	10 (77)
	Free-response information on conflicts	8 (62)
	Free-response on social interactions	7 (54)
**Please circle all the features you would like most from an application like RealLife Exp**		
	Connection to a community	6 (46)
	Connection to a care team	8 (62)
	Helpful information about symptoms	11 (85)
	Personal insights about behavior	11 (85)
	Rewards and badges for survey completion	7 (54)
**Please check the one feature you would like most from an application like RealLife Exp**		
	Connection to a community	0 (0)
	Connection to a care team	5 (38)
	Helpful information about symptoms	4 (31)
	Personal insights about behavior	3 (23)
	Rewards and badges for survey completion	1 (8)

### Implementation Costs

Forty-seven percent (29/61) of EP clients used a study phone, the majority of whom (n=26) were in county/state-funded clinics. The breakdown of study phones by clinic was as follows: 2 in EDAPT, 18 in SacEDAPT, 1 in Napa, and 8 in Solano. Two EP clients used a parent’s smartphone to participate in the study and 1 used a compatible Apple iPad device.

Costs to keep a smartphone line active for a month were US $17 per line. The Kyocera Hydro XTRM and Hydro Life phones cost US $149.99 per phone, whereas the Samsung Core Prime and On5 phones were US $139.99. Overall, the cost to provide smartphones to EP clients was US $7232.32; on average, the cost per client for the 5-month period in the study was US $249.39.

Research staff and LifeData technical staff provided technical support throughout the protocol. For this trial, research staff spent an average of 26 hours a week working on EP client and provider support, including recruitment, enrollment, and study-end appointments, and addressing technical glitches in the app and dashboard. The total cost for LifeData (including technical support and utilization of the app and dashboard) for the duration of data collection was US $4483.30.

## Discussion

### Principal Findings

This paper details a protocol for implementing mHealth technology in community outpatient EP clinics and reports initial feasibility data. Sixty-one EP clients (56% of the 108 eligible individuals) and 20 treatment providers (100% of eligible providers) enrolled in the study. Only 3% (2/61) of EP clients withdrew from the protocol due to lack of interest in participating and no providers withdrew, demonstrating significant commitment to using mHealth technology in treatment on the part of both clients and providers. These enrollment and dropout rates are comparable to our previous study (53% enrollment; 5% dropout) [10], in which participants received monetary compensation for completing surveys and staying in the study each month. This indicates that monetary incentives for completing surveys is not a factor in whether EP clients choose to enroll and remain in an mHealth technology protocol for up to 5 months as part of their treatment. Although survey completion rates in this study (~40%) are lower than those observed in our previous study (~70%) [10], ~40% survey completion over the course of 5 months in the absence of monetary incentives is encouraging, particularly in light of the frequent technical challenges posed by the app itself. This is a solvable problem for the field; we posit that with improved user experience and technical stability of an mHealth platform, survey completion will be higher. Future studies will directly test this hypothesis. Additionally, the finding that, in the 41 EP clients who completed the study, weekly survey completion was associated with negative and agitation/mania symptoms suggests that individuals with more severe symptoms may need additional support for successful integration of mHealth technology into their outpatient care. One way to achieve this could be to incorporate family/caregivers into the protocol, both in terms of supporting the client in completing surveys and for completing their own observational symptom ratings via smartphone.

All eligible treatment providers enrolled in the study, demonstrating significant interest in incorporating new technologies for enhancing care. Although the study was originally intended only to include therapists, interest by other treatment team members led to an expansion of enrollment to include psychiatrists across the sites. Similarly, preliminary data indicate that, when used as part of regular treatment sessions, survey data from EP clients were moderately useful in informing treatment decisions and addressing client needs. This level of interest, agreement to participate, and endorsed usefulness of dashboard data suggest an openness across provider roles to applying this type of technology as a part of routine EP care in the future. This is important because successful implementation and dissemination of mHealth technology as part of EP care will rely on provider uptake as well as client participation. Future research will need to address how to increase provider uptake and evaluate the impact of provider engagement with mHealth technology on client outcomes.

Our data indicate that a significant portion of participants (52%), particularly those in county/state-funded CMHCs, do not possess a smartphone that can support mHealth apps. Successful integration of mHealth technology will likely require budget consideration of the costs of providing smartphones and accompanying cell plans to a portion of clients for the duration of services (2 years for most EP programs). Although the cost per client is relatively low (approximately US $1250 per client for 2 years), this is a financial burden to CMHCs that will need to be offset by benefits such as increased billing productivity, reduced costs of care, and reduced rates of chronic disability. Additional costs will also need to be adjusted for, such as the cost of additional staffing support necessary to implement and integrate mHealth technology into standard clinic protocols. Cost-benefit analyses of the impact of implementing mHealth technology in EP care will be addressed in future publications.

An important question for smartphone technology implementation research is how to increase client enrollment. Results show that 56.5% (61/108) of clients across the 4 clinics were willing to participate in the study and 67% (41/61) of enrolled clients completed the study. Within each clinic, approximately half of eligible individuals enrolled, regardless of clinic capacity, location, and type. While this level of engagement and completion is meaningful, successful dissemination and integration of mHealth platforms in CMHCs will require higher enrollment rates. In this study, 3 key factors impacted enrollment: notifying clients of the research opportunity without violating privacy, staff turnover, and technical glitches. Given that this technology was implemented as part of a research study, recruitment heavily relied on the support of participating treatment providers at the clinics to inform clients of the protocol before research staff were able to approach the client. It is possible that this limitation on enrollment would be removed if the mHealth platform was introduced as part of standard clinical practice during intake procedures. That is, if mHealth technology is introduced entirely independent of research, with no additional appointments and requirements to liaise with research staff, these barriers might not exist. Additionally, there was significant turnover of treatment providers during the course of the study; both the UC Davis and Aldea Solano clinics experienced staff turnover during the recruitment period. This resulted in delays in scheduling clients and increased time between treatment sessions. Unfortunately, this negatively impacted client retention in the study, as affected clients oftentimes stopped responding to study outreach. Finally, difficulty with the mHealth platform (eg, failed notification deliveries, screen freezes, repeated forced log-outs) also likely impeded client engagement with the study, survey response rates, and satisfaction with the platform. Participants and treatment providers often required research staff aid to resolve technical glitches and engagement with the protocol was halted until technical glitches were resolved, causing frustration. A stable, user-friendly app, combined with real-time, in-house technical support and a clear protocol for resolving technical issues will be necessary for successful integration of mHealth platforms in community-based EP care.

Barriers regarding staff turnover and technological difficulties are ones that will likely affect implementation of a similar protocol in outpatient community settings, regardless of the relationship to research. Effective implementation of mHealth technology in the context of these barriers likely requires additional staffing/person-hours, such as a client-technology liaison, and technical support to counter the challenges of staff turnover and technology glitches. EP clients also made additional suggestions for improvements to the app that might increase engagement and satisfaction, including enhancing the user experience (eg, inclusion of visual summaries of survey responses) and increasing the flexibility of user engagement with the app (eg, increased variety of survey items, increased flexibility in response times). Similarly, providers suggested improved technical stability and dashboard enhancements that could facilitate greater incorporation of the platform during treatment sessions. Future studies attempting to use this technology in EP care should prioritize a flexible user-interface that presents accessible summaries of user data, on both provider and client ends, and technological support staff to ensure highest satisfaction and usability.

### Limitations

Four key limitations must be acknowledged. First, because this study only sought to establish feasibility of implementing a smartphone app in community outpatient care settings, rather than determine treatment efficacy, a control group was not included. To test treatment effects, future studies will need to include a treatment as usual control condition (ie, no mHealth add-on tool). Second, the shorter enrollment period at the Aldea sites restricted enrollment rates in those clinics due to limited research staff resources, highlighting the need for adequate staffing for such a protocol. Third, because individuals who declined to participate did not consent to research, we are unable to assess clinical or demographic factors associated with not consenting to use smartphone technology as part of clinical care. Finally, although many of the survey questions used in this protocol are broadly applicable across mental health diagnoses (eg, anxiety, depression, medication adherence, social interactions), future work is needed to determine the generalizability of similar platforms across a variety of behavioral health populations and care settings.

### Conclusions

These results provide preliminary data to support 3 conclusions: first, use of smartphone technology in EP outpatient clinics that are unaffiliated with a research center appears feasible; second, treatment providers are amenable to implementing smartphone technology into EP treatment protocols; and third, EP clients are willing to use smartphone technology as part of their care without reimbursement for survey responses. While this suggests that implementing smartphone technology is achievable and desirable in CMHCs, it is also important to highlight the importance of adequate staffing and technical support. Future studies must evaluate optimal methods of meeting this requirement while maintaining appropriate returns on investment in mHealth technology.

## Abbreviations

BPRS: Brief Psychiatric Rating Scale
CMHC: Community Mental Health Center
EDAPT: Early Detection and Preventative Treatment
EP: early psychosis
MD: Medical Doctor
MFT: Marriage and Family Therapist
mHealth: mobile health
MSW: Master’s in Social Work
PhD: Doctor of Philosophy
PsyD: Doctor of Psychology
SOAR: Supportive Outreach and Access to Resources
UC: University of California
